# Casein Micelles as an Emerging Delivery System for Bioactive Food Components

**DOI:** 10.3390/foods10081965

**Published:** 2021-08-23

**Authors:** Uzma Sadiq, Harsharn Gill, Jayani Chandrapala

**Affiliations:** School of Science, RMIT University, Bundoora, Melbourne, VIC 3083, Australia; s3820492@student.rmit.edu.au (U.S.); harsharn.gill@rmit.edu.au (H.G.)

**Keywords:** casein micelles, encapsulation, bioactives, microencapsulation, nano emulsion, hydrogels

## Abstract

Bioactive food components have potential health benefits but are highly susceptible for degradation under adverse conditions such as light, pH, temperature and oxygen. Furthermore, they are known to have poor solubilities, low stabilities and low bioavailabilities in the gastrointestinal tract. Hence, technologies that can retain, protect and enable their targeted delivery are significant to the food industry. Amongst these, microencapsulation of bioactives has emerged as a promising technology. The present review evaluates the potential use of casein micelles (CMs) as a bioactive delivery system. The review discusses in depth how physicochemical and techno-functional properties of CMs can be modified by secondary processing parameters in making them a choice for the delivery of food bioactives in functional foods. CMs are an assembly of four types of caseins, (α_s1_, α_s2_, β and κ casein) with calcium phosphate. They possess hydrophobic and hydrophilic properties that make them ideal for encapsulation of food bioactives. In addition, CMs have a self-assembling nature to incorporate bioactives, remarkable surface activity to stabilise emulsions and the ability to bind hydrophobic components when heated. Moreover, CMs can act as natural hydrogels to encapsulate minerals, bind with polymers to form nano capsules and possess pH swelling behaviour for targeted and controlled release of bioactives in the GI tract. Although numerous novel advancements of employing CMs as an effective delivery have been reported in recent years, more comprehensive studies are required to increase the understanding of how variation in structural properties of CMs be utilised to deliver bioactives with different physical, chemical and structural properties.

## 1. Introduction

Bioactive food components have received remarkable attention in developing functional foods and nutraceuticals due to their countless physiological health benefits. However, these bioactive components are rapid to inactivation and degradation by light, pH and temperature [1,2]. This rapid degradation can be dodged or slowed down by the encapsulation process till the absorption of these components at the targeted sites. Various encapsulation procedures have been projected to make bioactive components fully functional by preventing their chemical degradation during preparation, storage and transport [3]. There are four delivery systems (lipid-based, carbohydrate-based, hybrid system, protein-based) proposed based on processing conditions, physicochemical stability, sensory and nutritional properties of bioactive components [4,5].

A lipid base delivery system has been used to deliver lipophilic bioactives, by producing structured emulsions through physical modifications. These types of formulations have variable size ranges (5–100 nm), physical states, structural organisations and thermodynamic stabilities [6,7]. Carbohydrate-based delivery systems have been widely used for the protection of hydrophobic bioactives including fatty acids, aroma compounds and polyphenolic components, by protecting their dissolution in the stomach. Its constituents (mono, oligosaccharides) can be used as the encapsulating matrix, while the structural versatility of polysaccharides makes them ideal for targeted and controlled release of these bioactives by controlling pH, transit time and enzymatic degradation [8]. Mixed delivery systems have been introduced to overcome the limitations of food grade delivery systems due to their structural complexity and restricted properties of carrier bioactives by harnessing various interactions such as protein–polysaccharides interactions and electrostatic biopolymer interactions [9]. Proteins’ chemical and structural versatility makes them ideal for delivery of both hydrophilic and hydrophobic bioactives [10]. Indeed, the protein-based delivery systems have been considered a cheap and easy way to deliver bioactive components due to their porous structure, self-assembling nature, water binding abilities and surface-active properties [11].

Moreover, the choice of a reasonable protein for a specific transporter relies on the properties of the particle (e.g., size, charge, surface qualities and biodegradability), properties of the bioactive compound to be encapsulated (e.g., polarity, solubility and stability), and environmental conditions (e.g., pH, ionic quality, solvent properties and temperature) [12]. Though various proteins have been widely used as delivery vehicles, milk proteins (caseins and whey) are exotic encapsulation particles due to their elastic structural and functional properties. They have efficient bioactive binding abilities, better encapsulation efficiencies and controlled and target release of bioactive components [3]. As compared to whey proteins, casein micelles are recognised as a natural vehicle for bioactive components since casein proteins have a porous structure with cavities and are recognised as GRAS (Generally Recognized as Safe) [13]. Casein micelles have unique structural and physicochemical properties, such as binding with ions and small molecules to form macromolecules, exceptional stabilising characteristics, self-assembling, emulsifying and water-binding abilities. The porous structure and unique functional properties make them appropriate for the transport of bioactive components; therefore, they have been used in traditional and new drug delivery systems [14].

Caseins are phosphorylated proteins, representing 80% (*w*/*w*) of all milk proteins present in milk. The caseins are synthesised in the mammary glands, secreted into vesicles and exported through exocytosis [15]. They are joined through hydrophobic collaborations, hydrogen bonds, electrostatic interactions and, calcium phosphate nanoclusters to shape as spherical structures [16]. Casein demonstrates high stability at temperatures > 100 °C and pressures up to 100 MPa [17]. However, it is immediately destabilised with aspartate protease’s proteolytic action or acidification, resulting in curd formation [18].

Casein micelles showed pH-dependent conducts, i.e., tightening at lower pH and swelling at higher pH values. Dropping the pH (4.6–4.8) below isoelectric point (Ip) results in aggregation of casein micelles with the release of calcium, signifying the great importance of accessibility of cations for casein micelle formation [19]. In contrast, increasing the pH promotes increased electrostatic repulsions between the casein molecules, resulting in the loosening of the micellar structure and thereby increase in size. As the human stomach pH is profoundly acidic yet neutral in the duodenum, the casein micelles’ pH-dependent actions can be favourable for the controlled release of bioactive components taken orally. Thus, casein’s pH-sensitive gel’s inflammatory behaviour makes it beneficial for the eternal release of bioactive components [20,21,22].

Furthermore, casein micelles are amphiphilic, which then can act as a nano-vehicle for both hydrophobic bioactive components such as vitamin (D_2_, D_3_, E, K) and/or hydrophilic macromolecules such as whey protein and polysaccharides. The vulnerability of caseins to proteolysis [23] guarantees the high discharge of bioactives by a proteolytic enzyme in the gastric tract. The cellular uptake investigation of casein micelles revealed that casein spheres could enter the plasma layer in an independent energy fashion due to the proline-rich peptide sequence in casein [24]. Moreover, caseins have various preservation capabilities essential for the safety of sensitive encapsulated bioactive components, thereby controlling these bioactive agents’ biosafety and bioavailability. The casein spheres could significantly advance as one of the best nutraceuticals and drug delivery systems due to its protein matrix rich in surface reactive groups, hollow structure and innovative cell-penetrating properties [14,25].

Although much work has been done regarding caseins as a delivery system for pharmaceuticals, functional foods and nutraceuticals [26,27,28,29], still some areas such as induced structural modification of casein micelles, by altering secondary processing parameters, need to be explored. A recent review by Nascimento and colleagues [30] presented an overview of casein-based hydrogels. Ranadheera [31] examined casein and casein micelles’ unique properties as capsules, emulsions, hydrogels and film coatings and observed that different processing parameters can alter casein micelles’ techno-functionalities, consequently facilitating the encapsulation of food bioactive components inside casein micelles by binding at its hydrophobic and hydrophilic domains. Thus, this review provides updated and most recent studies about casein micelle as a delivery vehicle with particular attention to deliver bioactives in functional foods and nutraceuticals, along with detailed facts on how pH and temperature affect the incorporated food bioactive component’s binding and release properties.

## 2. Casein Micelles and Its Structure

In milk, caseins are generally present as casein micelles. Protein accounts for about 95% of the dry matter in casein micelles, with the rest being minerals collectively referred to as micellar calcium phosphate (MCP). MCP is mostly composed of calcium and phosphate, with trace amount of magnesium, citrate and other minerals [32,33]. The average size of casein micelles of individual cows’ ranges from 50 to 500 nm, which remains consistent during lactations and across lactations. However, the change in size and polydispersity of casein micelles have been observed in milk obtained from different cows [34,35]. Casein micelles are exceptionally hydrated and hold around 3.3 g water per g of protein [36].

Casein micelles are supramolecular structures framed through the association of four fundamental caseins α_s1_, α_s2_, β and κ casein in different proportions as 40%, 10%, 35% and 15% (w.w), respectively [37]. Each type of casein exhibits unique physicochemical properties, as shown in Table 1. The α_s1_, α_s2_ and β caseins are highly phosphorylated, showing phosphorylation at 8, 9–11, 5 serine (Ser) residues, respectively, which are vital for stabilising calcium phosphate nanoclusters in casein micelles. κ caseins are glycoproteins, showing little phosphorylation at Ser residue and the only casein showing phosphorylation at Threonine residues and does not precipitate at high concentrations of calcium ions [38]. These post-translational variations (glycosylation and phosphorylation) increase the hydrophobicity of caseins to interact with phenolic compounds and to acts as a nanocarrier for bioactive components. The phosphoserine residues occur in clusters on specific serine residues (SER) specified as phosphoseryle clusters, which aids in creating a flexible, amphiphilic, hydrophobic and hydrophilic structure. Both α caseins have molecular chaperon characteristics, that prevents coagulation and precipitation as a result of heat by stabilising its unfolded targeted proteins through interaction [39].

β caseins’ intermolecular hydrophobic interactions and amphiphilic structure are responsible for its self-assembling property, which is ideal for drug delivery systems, while phosphorylation of β caseins enhances its GI digestibility [40]. κ caseins constitute the minor part of the casein micelle surface and provide a hydrophilic layer vital for the steric stabilisation of casein micelles by inter micellar electrostatic and steric repulsions [41]. Moreover, κ caseins bind to the hydrophobic calcium core of α_s_-β-caseinate to prevent aggregation by providing a solvated coat [42,43]. During heating and rennet cleavage, the κ caseins contribute dynamically in thiol-catalysed disulfide interchange reactions with whey proteins, promoting micelle coagulation. These κ caseins functions are governed by the protein’s three-dimensional structure on the micelle surface [44].

α_s1_ caseins are moderately hydrophobic proteins containing 25 positively charged and 40 negatively charged amino acids, 20 aromatic residues and some discrete patches of hydrophobicity compared to α_s2_ with 33, 39 and 20 positively, negatively and aromatic residues, respectively, with five regions of different hydrophobicity.

β caseins are strongly amphipathic, starting from low hydrophobicity with overall net charged amino acids (1–40) followed by moderately charged amino acids (41–135) and ends up at little hydrophobicity with high charged amino acids (136–209). κ caseins have negatively charged amino acids in the N-terminal region of 1–20 and C-terminal regions of 115–169, whereas positively charged amino acids comprise N-terminus regions of 1–116 generate hydrophobicity at 21–110. Glycosylation and phosphorylation in the C-terminal part increase hydrophilicity in addition to hydrophilic regions between 1–20 and 110–169 amino acids [45].

Another characteristic of caseins is the proline residues, specifically in β caseins, which disrupt the casein micelle structure and give a non-globular nature to caseins with an open structure. These proline-rich caseins carry numerous properties like resistance to heat denaturation, favouring the elastic conformations in solution, great structural flexibility against environmental stresses, specific proteolytic cleavage and targeted drug delivery [46].

### 2.1. Casein Interactions

Numerous studies have been undertaken on how caseins interact with each other in the past. In 1920, it was considered that caseins undergo self-association as well as with other caseins. The association of caseins within a micelle depends on pH, ionic strength and temperature [47]. Von and Waugh [48] were the first to perform a thorough study about caseins interactions and the complexes they may form when calcium concentrations, temperature and pH are varied [48]. However, there have been several divergent opinions and debates about the critical forms of relationships that dictate casein structure [46].

#### 2.1.1. Self-Association of Caseins

All types of caseins can self-associate into dimers, tetramers and hexamers [49]. However, the association of α_s1_ casein is more extensive than α_s2_ caseins. Self-association of α_s1_ casein takes place into dimers, tetramers and hexamers at pH 6.6, whereas raising the ionic strength at the same pH results in a decrease in α_s1_ casein monomers and a rise in oligomers [50]. At increased pH values up to 6.6 improves electrostatic repulsion and decreases interaction [51]. Additionally, differences exist due to genetic variance, with variant C of α_s1_ casein exhibiting somewhat greater self-association than variants B and D [50]. Due to the temperature dependency of the self-association of α_s1_ caseins, only dimers are found at 37 °C, but higher-order structures are formed at lower and higher temperatures [52]. In contrast, α_s2_ caseins at 20 °C and an ionic concentration of 0.2–0.3, the interaction is maximal and declines at higher and lower ionic concentrations. α_s2_ casein is spherical at 20 °C. However, when α_s2_ casein is incubated at 37 °C and 50 °C, it forms ribbon-like fibrils of 12 nm diameter and greater than 1 µm long loop shape structures [53,54].

β caseins exhibit temperature-dependent micellisation, with the C-terminal region serving as the centre of the micelles, due to the existence of distinct polar and hydrophobic regions. The β casein occurs mainly as monomers below 5 °C, although certain polymers are available as well. With increasing temperature, β casein undergoes self-association reactions, resulting in micelles with a small size distribution. Micelles tend to form at a critical concentration, which varies from 0.5 mg/mL to around 2 mg/mL, based on temperature, ionic pressure and pH. The micelles contain almost 15 and 60 monomers and a radius between 8 and 17 nm. [55,56]. The increased ionic strength of β caseins changes the monomer–polymer balance towards polymers but has no impact on the number of monomers in the micelle. At the same time, rising temperature shifts the equilibrium towards polymers and raises the number of monomer units in the micelle. A shell model for the polymer micelle can be used to characterise the features of this monomer–polymer equilibrium, with a continuous range of intermediates between the monomer and the biggest polymer micelle [57].

κ caseins form multimeric complexes of molecular weights of 1180 Da at 25 °C and 1550 Da at 37 °C with a radius of 5–10 nm and these values remain constant irrespective of the protein contents [58]. The disulphide bridges between κ caseins are reduced (reduced κ caseins), resulting in amphipathic monomer units that can form micellar structures as in β caseins. However, micellisation of this reduced κ casein does not exhibit a significant temperature dependency, meaning that hydrophobic interactions regulate the relationship of reduced κ casein to a lesser extent than β casein. At an ionic pressure of 0.1 and 1.0, the critical micelle concentration is 0.53 mg/mL and 0.24 mg/mL respectively [59].

Micelles of reduced κ casein were reported to comprise 30 casein molecules with molecular weights ranging between 570 and 600 kDa and diameter ranging between 21 and 23 nm [60]. Reduced κ casein also formed fibrillary structures with a 10–12 nm diameter and a length of 600 nm when heated to 37 °C [61]. When native κ casein is used, its dissociated form is the one that participates in fibril synthesis. This fibril formation has been shown to raise the ratio of β sheet structure is more extensive at higher temperatures and more extensive for non-glycosylated κ casein than for its glycosylated version [54,62].

#### 2.1.2. Interactions with Other Caseins

Aside from self-association, all caseins tend to interact with other caseins. These associations are essential for the formation of casein micelles [38,63]. At 37 °C, α_s1_ casein seems to form only dimers but no monomers [52] and β casein forms very large micelles comprising an average of 20 molecules when alone. However, when α_s1_ and β caseins are combined at the ratio of 1:1 like they are in milk, smaller micelles are formed [38]. This indicates the development of mixed complexes and displaying dominancy of α_s1_ casein–β casein complexes formation over self-association of β casein [38]. Similarly, in the mixture of α_s1_ and κ caseins in the ratio of 4:1, as they occur in milk, the presence of α_s1_ casein disrupts the giant κ casein micelles. Hence, it gives the idea that interactions amongst caseins remain extremely cooperative and favourable and interactions between κ and α_s1_ caseins are stronger than that amongst α_s1_ and β caseins. However, α_s1_ and β casein interactions are thought to be hydrophobic and hydrophobic interactions are week interactions [63].

#### 2.1.3. Hydrophobic Clustering of Caseins

Hydrophobic interactions occur when two opposing surfaces come close together by the exclusion of water. Only the interactions of β caseins with other caseins are typically hydrophobic, which results in β casein dissociation from casein micelles when hydrophobic interactions are minimised in milk upon cooling [63]. Typically, when β casein is cooled, up to 30% of it dissociates from the casein micelles, while the remainder remains attached to the micelles. However, when milk is heated at 30 °C, all dissociated β caseins reassociate with the micelles. This happens to β caseins associated with other caseins rather than attaching to calcium phosphate nanoclusters [64]. Although certain β caseins will dissociate from the casein micelle, this does not seem to disrupt the casein micelles structure. As other caseins do not form hydrophobic interaction, so there is no dissociation upon cooling. According to amino acid concentrations, 28% of κ casein, 30% of α_s2_ casein, 32% of α_s1_ casein and 34% of β casein residues are hydrophobic, or about 1 in 3 [51,65]. Hydrophobic Clustering Analysis (HCA) was carried out by Horne, 2017 to explore the hydrophobic residues along the caseins sequence. The sole purpose of 2D-HCA was to show that all caseins contain segments that might interact hydrophobically with other caseins [46].

**Figure 1:**
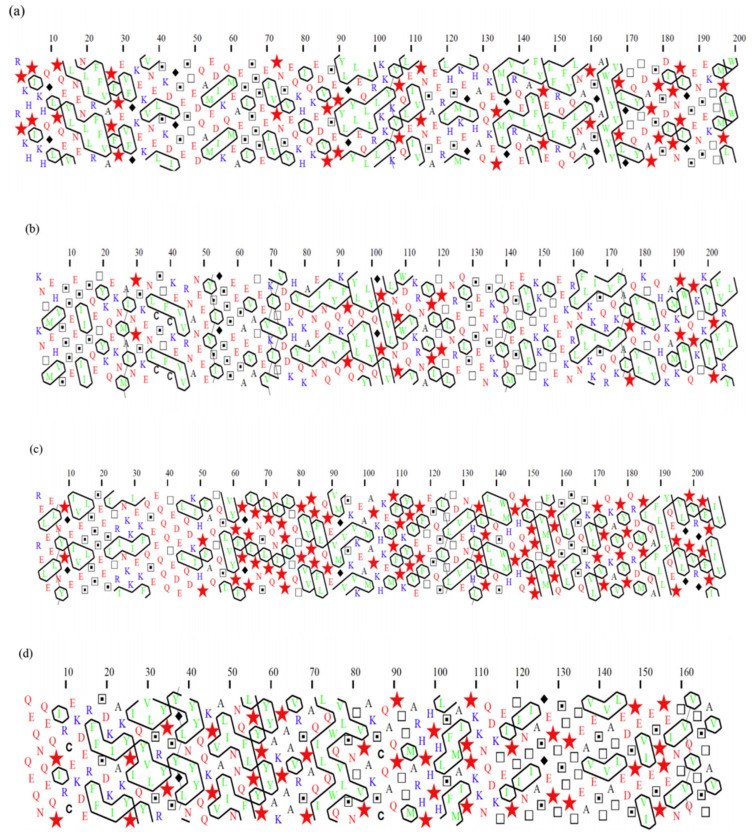
Hydrophobic cluster analysis (HCA) of (**a**) α_s1_ casein, (**b**) α_s2_ casein, (**c**) β casein and (**d**) κ casein, shown as 2D-HCA plots, proline has been shown by symbol star, Glycine by diamond, Serine by dotted squares and Threonine by squares. Reproduced with permission fromAdapted [Lucey, J.A.; Horne, D.S.], [Perspectives on casein interactions], [Int. Dairy J.], (2018).

Additionally, the 2D-HCA plots illustrated clustering of the serine groups in isolated fragments of these proteins (represented by dotted squares in Figure 1). In α_s1_ casein, for example, the phosphorylated serines are found between residues 45 and 89, and Figure 1a depicts two separate clusters of these serine residues. Phosphorylation increases the hydrophilicity of the areas containing these serine units, thereby reducing the possibility of developing hydrophobic clusters near these phosphorylated clusters. Fortunately, unlike the primary hydrophobic clusters, the phosphoserine clusters in caseins are distributed throughout the chain (Figure 1). For instance, in α_s1_ casein, multiple unique hydrophobic regions may be observed (i.e., sections containing numerous broad hydrophobic clusters amongst residues 1–44, 90–113 and 132–199) (Fia in agreeing to previously released data) [36]. These plots were also compatible with the previous classification where caseins are thought to be an unordered structure as intrinsically unstructured proteins (IUP) and later recognised as IUP with highly hydrophobic regions [66] to self-associate in the absence of calcium phosphate [36]. According to Farrell and colleagues in 2006 [67], caseins can be called intrinsically unstructured proteins (IUP). However, these scientists in 2012 [68] reported/suggested that although certain features of caseins fit it to classification as IUPs, contrary to other IUPs, casein regions are very hydrophobic (Figure 1), resulting in a greater propensity for self-association. Additionally, all of these statements about potential IUP states are for isolated caseins without calcium and phosphate additions [69]. As a result, it is unclear how applicable IUP state predictions are for caseins during micelle assembly when both calcium and phosphate influence the caseins’ interaction behaviour.

It has been hypothesised that high proline contents of caseins are responsible for their flexibility and non-globular nature. Proline residues are widely regarded as disruptors of secondary structure in HCA [70] that are shown as stars in the 2D-HCA plots in Figure 1. Arunachalam and Gautham studied hydrophobic clusters in 781 protein structures. They found a clear preference for (recognised) hydrophobic amino acid residues to form hydrophobic clusters, but hydrophobic groups from hydrophilic amino acids (like CH2) may also take part in cluster formation [65]. Horne noted some nonpolar residues along with charged interaction and concluded that casein micelles are assembled by calcium–casein bindings through colloidal calcium phosphate [46].

#### 2.1.4. Casein–Mineral Interactions

Casein–mineral interactions are critical in the formation, stabilisation and functional properties of casein micelles [45]. The binding of calcium to casein is relational to their phosphoserine residues, and dephosphorylation significantly decreases this bonding [69]. Swaisgood [37] claimed that the binding of ions, especially calcium to casein disrupts the equilibrium of electrostatic forces and hydrophobic interactions, which manifests itself in the self- or inter-association of caseins. These associations are primarily between the negatively charged side groups of amino acids and the casein cations, specifically the carboxylate groups of Glutamic acid and Aspartic acid and the phosphate group of Serine phosphate. Because of the poor solubility of calcium and magnesium phosphates, these salts easily interact with the Serine phosphate group of caseins when present in solution form. Interactions of calcium and magnesium with Glutamic acid, Aspartic acid and Serine phosphate residues reduce the casein’s net negative charge, resulting in a lack of solubility and facilitating the interactions between α_s1_, α_s2_ and β caseins, which are referred to as calcium-sensitive caseins. κ casein, on the other hand, is not vulnerable to Ca-induced solubility loss and may potentially help stabilise the other caseins against calcium-induced loss of solubility [71].

Caseins’ ability to bind calcium is highly conditional and decreases as pH declines and ionic strength increases. Almost all Serine phosphate residues in milk are associated with calcium or magnesium, and P-NMR analysis revealed few or no free Serine phosphate residues [72]. Dephosphorylation of casein, which may be accomplished enzymatically or by heat treatment or exposure to alkaline environments, significantly decreases the calcium-binding of caseins [73]. Caseins can inhibit calcium phosphate precipitation in addition to bind calcium and magnesium ions [74]. Detailed studies revealed that the centers of phosphorylation, identified as at least three Serine phosphate residues, found in α_s1_, α_s2_ and β caseins have the potential to adsorb onto the surface of calcium phosphate structures and inhibit further growth [75]. This mechanism is analogous to how caseins are thought to stabilise nanoclusters of amorphous calcium phosphate inside casein micelles.

At milk pH, all caseins carry a net negative charge, so regarding their interactions, the importance of this cannot be overemphasised. The charge on caseins governs their association equilibria; as sensitivity to pH and ionic strength testify. Calcium-binding may reduce this charge, resulting in the precipitation of α_s_ and β caseins. The kinetics of precipitation can be shown to be a property of Q^2^, where Q is the net charge after accounting for Ca^2+^ ions attached to each casein molecule. Calcium-binding may also minimise the net micellar charge, and it has been shown that the temperature dependency of this binding is responsible for the remarkable decrease in the rate of rennet coagulation [70]. All these observations highlight that casein–casein interaction and casein mineral binding can play an important role in facilitating casein micelles structure.

#### 2.1.5. Models of Casein Micelles

**Figure 2:**
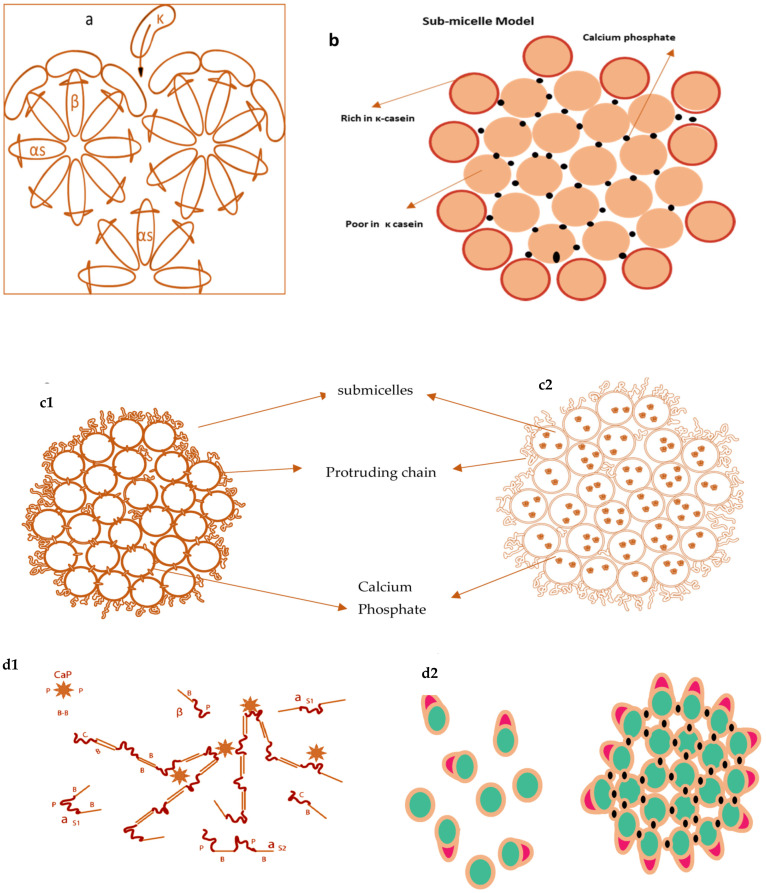
Casein micelles model by Waugh 1958 (**a**), models by Schmidt in 1982 (**b**), model proposed by Walstra in 1990 (**c1**) & 1999 (**c2**), (differs in calcium phosphate location), Dual binding model by Horne (2003) (**d1**) and interpretation of Schmidt’s model in 2005 (**d2**).

Several models of casein micelles have been reported based on the characteristics and interactions of caseins. The oldest model was the core coat model proposed by Waugh, 1958 as in Figure 2a. According to this model, casein micelles composed of variable-sized cores of insoluble slats of α or β casein covered by a coat of κ caseins [76]. Later, a submicelle model was projected by Schmidt, 1982 shown in Figure 2b, which suggested that casein micelles were distinct subunits composed of colloidal calcium phosphate crosslinkages [77]. Walstra in 1984 proposed a submicelle model according to which casein micelles are the assembly of roughly spherical subunits or submicelles held together by hydrophobic interactions and calcium phosphate bridges [78].

One submicelle is formed by α and β caseins having hydrophobic regions in the centre and another submicelle is composed of α and κ casein having more hydrophilicity. κ casein forms the hairy layer outside the submicelle and will hinder the additional accumulation of submicelle by steric and electrostatic repulsion [78]. Walstra (1999) (Figure 2c) advocated that casein micelles are spherical proteinaceous particles glued together by the CCP with variability within the micelles and between the micelles [78]. In the 1990s, two novel models were proposed by Holt [79] of discrete submicellar structure within casein micelles. Holt [79] proposed a polymerisation or gel network model for the casein micelle, in which development was envisioned because of interactions between phosphoserine clusters on calcium-sensitive caseins and CCP nanoclusters. According to this model, the development of these nanoclusters will promote micelle assembly via the spontaneous linkage of additional phosphoproteins. Since the α_s_ caseins were found to contain more than one phosphoserine clusters, network development will proceed. Later on, this model was termed as nanocluster model [79].

Horne’s dual binding model (Figure 2d) is an alternative approach [80,81] of previously reported internal structure models with slight refinement. According to this model, casein micelles assembly and growth proceed by two routes; hydrophobic interactions (rectangular bars) are responsible for casein micelles formation, whereas electrostatic repulsions (loops) limit the polymers’ growth. This mechanism allows branching and thus leads to a three-dimensional network holding enough water. This model outlines the interactions that may occur during micelle assembly but does not go into depth about the micelle’s actual internal structure. Initially, the model did not recognise the shape of the calcium phosphate, but more recent publications have defined calcium phosphate using a nanocluster model revision [82].

**Figure 3:**
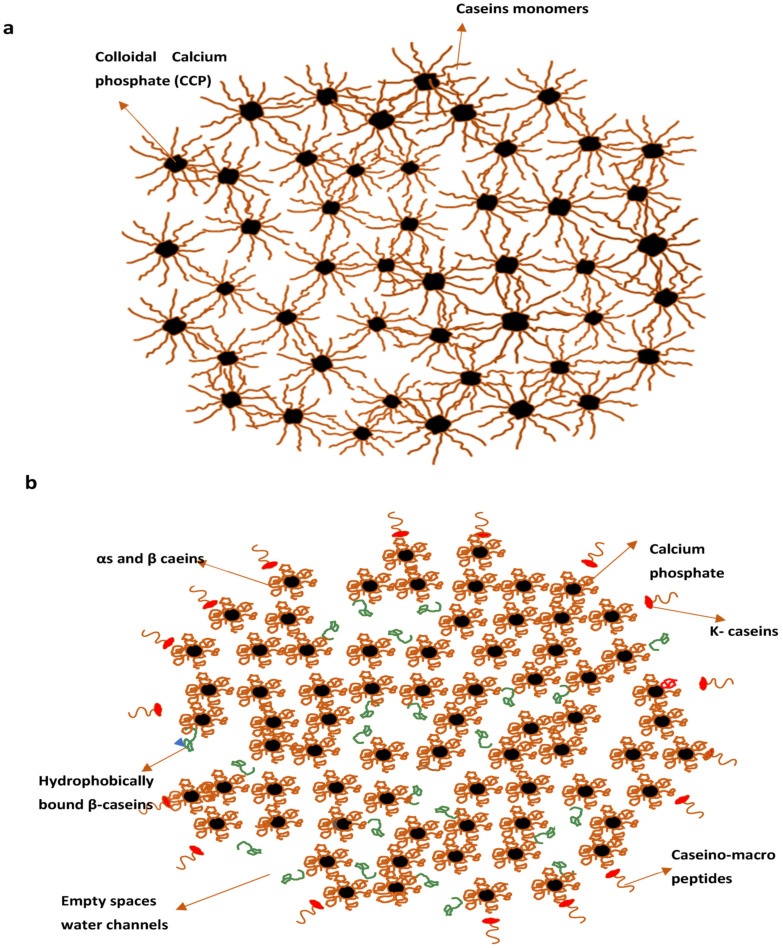
Casein micelles model by Holt (**a**) Casein micelles model by Dalgleish (**b**). Reproduced with permission from [Dalgleish, D.G.], [On the structural models of bovine casein micelles—Review and possible improvements], [Soft Matter], (2011).

The nanocluster model (Figure 3a), proposed by Holt, is more recent in which protein matrix (a nanogel) have distributions of colloidal calcium phosphate (CCP) nanoclusters as small “cherrystones” [71,75,83]. This model’s main features were the cementing character of the CCP, steric stabilisation by surface located κ casein and potential surface stabilisation at pH 6.7. Huppertz and colleagues recently introduced the subunit model, in which protein subunits bound by nanoclusters of PCP (Primary Casein Particles).This seems identical to the earlier Schmidt form of a model developed from Schmidt’s work on preparing artificial micelles from sodium caseinate and sodium salt mixtures [36].

In general, the micelle core can be easily modelled using PCPs composed of four α_s1_ and β casein molecules and one α_s2_ molecule that interact with a three-dimensional network by crosslinking through calcium phosphate nanoclusters (Figure 3b). When the micelle’s centre is viewed as a network of non-spherical primary casein particles linked by calcium phosphate nanoclusters, it is obvious that the protein density would not be uniform throughout the micelle; rather, regions of high and low protein density exist [18,35]. It also implies that the micelle has regions with high and low moisture contents. Protein-rich domains would be comparatively dehydrated, with predominant hydration of 0.5 g water×g^−1^ casein. However, the average hydration of casein micelles is significantly enhanced by aqueous gap spaces within PCPs within the micelles and the hydration of the casein micelle surface. The connectivity of the water-rich domains in Figure 3b given by Dalgleish resembles the water channels in casein micelles described by Huppertz later [18,36].

## 3. Factors Affecting Techno-Functionalities of Casein Micelles

After discussing the numerous mechanisms by which caseins interact during micelle formation, it is important to explore how such interactions can be engineered to change the functional properties of casein micelles. The structures and techno-functionalities of casein micelles could be modified by various intrinsic and extrinsic factors as shown in Table 2. However, this review will focus just on temperature and pH effects.

### 3.1. Effect of Temperature on Techno-Functionality of Casein Micelles

The heating of proteins induces conformational changes, exposing the hydrophobic sites. Owing to the absence of a tertiary structure, casein micelles are heat stable. However, distinct changes have been noted concerning the frequency of the heat. Several biochemical modifications are identified, including deamidation of asparagine and glutamine residues, proteolysis [84], and reticulation between amino acids, which results in protein polymerisation, disulphide bridge breakdown and exchange of free thiols on cysteine residues. During heat treatment, the mineral fraction, especially calcium phosphate, becomes less soluble in the aqueous phase, which may interact with casein micelles [85]. When the temperature is less than 95 °C for a few minutes, the changes in salt equilibria are reversible. In comparison, prolonged exposure to high temperatures (for example, 120 °C for 20 min) results in irreversible alterations to the casein micelles and salt distribution. Casein phosphoseryl residues may be partly hydrolysed at temperatures greater than 110 °C [86]. There are limited and old dated reports describing the physicochemical changes in casein micelles induced by cooling.

Koutina and colleagues [87] reported that calcium and phosphorus concentrations in the soluble phase were more significant at 4 °C than at 40 °C due to the increased solubility of calcium phosphate at lower temperatures. Simultaneously, reduced hydration of casein micelles and release of β casein from the micellar structure has been observed [64]. Indeed, temperature reduction alters protein interactions, which allows the transfer of β casein into the aqueous system. These modifications are reversible, and the prior clustering may be restored after heating; however, the native framework is not fully restored because β casein would not revert to its original location [88]. Liu and colleagues [88] confirmed that the volume of soluble casein, hydration and apparent voluminosity of casein micelles reduced as the temperature increased demonstrating that casein micelles structure and mineral in milk were temperature-dependent between 10 °C and 40 °C. However, the mineral system reaction is prompt during this heating, while casein micelle re-equilibration occurs gradually during cooling. This method could be opted to obtain purified β casein and obtain remained novel casein micelles (less mineralised, depleted in β casein and more hydrated) with innovative techno-functionalities [89].

### 3.2. Effect of pH on Techno-Functionality of Casein Micelles

pH is the most critical factor affecting the functionality of casein micelles. Casein molecules’ structure and charge are pH dependent. Low pHs decrease their ionisation and alter intra and intermolecular connections. Thus, when caseins are acidified, the phosphoseryl residues and carboxyl groups undergo an ionisation state transition, owing to their propensity for protons and their protonation is determined by their pKa values. Thus, casein molecules are negatively charged at neutral pH. They bind protons during acidification and casein molecules get less and less negatively charged before reaching their isoelectric pH. At this pH, casein molecules aggregate and have negligible solubility [95]. During acidification, caseins’ organic and inorganic phosphate, citrate and carboxylic residues get more protonated (or less ionised). Simultaneously, the aqueous phase has become less saturated with calcium phosphate because of its dissociation. Therefore, the micellar calcium phosphate dissolves gradually as the accumulation of calcium and inorganic phosphate in the aqueous phase rises [95,116]. In the aqueous phase, dissociation of casein molecules occurs, and the degree of dissociation between minerals and casein is pH-dependent. A fraction of calcium and the whole inorganic phosphate is dissolved at pH 5.2. Calcium is completely soluble at pH 3.5 [116]. Simultaneously, when casein molecules become less charged, their interactions with other caseins and with water are changed. κ casein’s hairy coating contracts and subsequently collapses as a result of acidification. As a consequence, the steric stability of casein micelles reduces resulting in appealing interactions between micelles [94].

At pH 5.6, casein micelles enlarge and dissociation of caseins approaches a plateau, with β casein dissociation reaching a maximum [96]. A new limited group of caseins similar to casein aggregates is found in this pH spectrum of 5.6 to 6 [97]. These smaller units range in diameter from about 20 to 35 nm and have a molecular weight of 106 and 107 g·mol^−1^. As the pH value decreases (6.7, 6.4, 6.1, 5.8, 5.5), the proportion of these smaller particles increases. The non-dissociated casein micelles seemed to be close to native casein micelles in size, hydration, appearance and zeta potential [97]. Demineralisation of casein micelles by reducing the pH from 6.7 to 5.2 resulted in a reduction of micelles’ granularity as determined by cryo-transmission electron microscopy, atomic force microscopy [126], and by the presence of a distinctive point of inflection in SAXS profiles [127]. At pH 4.6, caseins have no charge and therefore have negligible solubility and got precipitate. Acidification causes a similar degree of micellar destruction regardless of the type of acid used (lactic, citric), as physicochemical modifications primarily depend upon pH. However, the composition of the aqueous phase, especially its ionic state, varies according to the acid form, which has an impact on the structure and functionality of acidified caseins [16].

Increasing the pH from 7 to 9 caused the inclusion of casein molecules in serum and the destruction of casein micelles [98]. Simultaneously, a reduction in calcium and inorganic phosphate concentrations in the aqueous phase were detected. According to Ahmad et al. [98], the inorganic phosphate ion shifts its ionisation status from HPO_4_^−2^ to PO_4_^−3^ at alkaline pH. Due to the increased affinity of this latter form of phosphate for calcium, a demineralisation of casein micelles was observed. Mineral concentration reductions in the aqueous phase can boost the solvent’s consistency and increase casein micelle dissociation by diminishing cohesive connections amongst casein’s hydrophobic sections [128]. Along with mineral shifts, Huppertz [99], hypothesised that variations in the ionisation of caseins might also lead to this micellar disturbance. Scientists explored a low-cost, low-energy and organic solvent-free encapsulation technology by utilising the pH-dependent solubility of curcumin and the self-assembling properties of sodium caseinate [129].

## 4. Casein Micelles–Based Delivery Systems

The scientific community has spent many decades attempting to characterise and comprehend the complexity of casein micelles in terms of composition, structure and functional properties. As discussed in the previous section, casein micelles may be modified under various temperature and pH conditions to alter their techno functionalities. However, other physical, chemical or enzymatic methods have also been used to alter the technological functionalities of casein micelles and these innovative micellar functionalities have been utilised in various functional foods and nutraceuticals as carriers for bioactive compounds. The bioactive’s low absorption and efficacy are associated with deprived bioavailability upon taking through the oral route and their vulnerability to degradation (chemical, physical and enzymatic) during different processing, storage and transportation. These factors require the protection of these bioactive. In this context, casein micelles were exploited to form microparticles, nanoparticles and hydrogels for targeted delivery of bioactive food compounds at the site of action, as illustrated in Table 3 [40,130,131,132,133].

### 4.1. Caseins as Micro and Nanoparticles

In the nutraceuticals industry, both the hydrophobic and hydrophilic properties of casein micelles have been exploited [166]. The hydrophobic molecules present several bonding options when binding to the caseins, for example, hydrogen bonding, van der Waals forces and hydrophobic interactions [23]. A hydrophobic molecule of vitamin D_2_ has been encapsulated by Semo and colleagues [14], within casein micelle by using sodium caseinate. However, the pH of the solution was changed to 6.7 according to natural milk pH. Caseins were able to encapsulate vitamin D_2_ efficiently due to hydrophobic domains and self-assembled micelle structure. Moreover, vitamin D_2_ was found 5.5 times more in casein micelles than in serum.

Researchers Cohen and Haham [145,167], reported that the significant protection is provided by the re-assembled casein micelles (r-CM) to the encapsulated vitamin D_3_ against degradation caused by heat and cold storage as compared to unencapsulated vitamin D_3_. They used light microscopy and UV absorbance spectra test to provide qualitative proof for the binding of vitamin D_3_ and caseins before and after freeze-drying and reconstitution with water. Vitamin D_3_-rCM before and after freeze-drying and reconstitution had a negligible effect on the size distribution, visual presentation and vitamin D_3_ concentration of the loaded r-CM. Encapsulated vitamin D_3_ was also observed to be slightly more protected than vitamin D_3_ dispersed in water under simulated gastric and upper-intestinal environments. The in vitro bioavailability of vitamin D_3_ using Caco-2 cells suggested poor absorption rates for both formulations. However, there was no significant difference with the absorption of vitamin D_3_ by Caco-2 cells of digested r-CM. Ultimately, this study was an addition to the evidence of the tremendous potential of r-CM for the efficient and safe distribution of responsive hydrophobic bioactive compounds in foods.

Encapsulation of epigallocatechin gallate (EGCG) inside recombined casein micelles from milk was trialled by Haratifar and Guri to protect it from oxidation [168]. Epigallocatechin gallate (EGCG) has hydrophilic features and is soluble in H_2_O. Only 0.1–1.1 per cent of the entire consumed EGCG is bioavailable for captivation with encapsulation. This poor bioavailability is the result of decreased chemical steadiness and decreased intestinal permeability [169]. They investigated that at concentrations > 0.03 mg of EGCG/mL in skim milk, the binding of EGCG to casein micelles had no impact on the bio efficacy of EGCG or cell absorption. In Vitro, anti-cancer studies indicated that the EGCG–milk complexes were capable of greatly inhibiting HT-29 cancer cell proliferation in a manner close to that of free EGCG (without encapsulation). However, the biological health benefits can only be benefited when EGCG are readily accessible and are in their active state within a food system [170]. This was achieved via encapsulation using rCM.

The recombined casein micelles (r-CM) application in the encapsulation of hydrophobic nutraceuticals were investigated by Zimet [171], and recently by Ghayour [27]. They examined the binding features of folic acid (FA) and EGCG to unfolded sodium caseinate (Na-caseinate). Casein micelles re-unite from sodium caseinate and could be utilised in a comparable mode to usual casein micelles. Additionally, the bioactive’s binding operation (entrapment) of EGCG is higher to casein and then r-CM instead of casein nanoparticles (CNPs). Hence, higher encapsulation efficacies (85%) are achieved in r-CMs depending upon pH, as pH increased resulted in the loosely bulk structure of casein micelles. Hence, r-CMs can be an excellent carrier for hydrophilic nutraceuticals; however, further research is required to check the bioavailability of these components in casein-based nanocarriers [147].

Researchers described curcumin encapsulation, a lipophilic phytochemical, in self-assembled casein nanoparticles (sodium caseinate NaCas) as function of pH. They evidenced the utilisation of NaCas nanoparticles for binding deprotonated curcumin at pH 12, where casein particles become porous by dissociation. However, by decreasing pH, efficient, low cost, chemical-free encapsulation of curcumin was noticed due to re-association of caseins. Curcumin encapsulated in casein nanoparticles indicated essentially improved anti-proliferation action against human colorectal and pancreatic disease cells. These types of low energy, low-cost nanoencapsulation techniques could be used in future to encapsulate lipophilic bioactives like phytochemicals into caseins for the development of innovative functional foods and nutraceuticals [129].

Moeller and colleagues [172] used casein micelles to encapsulate lipophilic components (β-carotene, vitamin D_2_ or docosahexaenoic acid (DHA) at pH 5.5 and temperature 2 °C. They investigated that recombined casein micelles were able to load β-carotene and vitamin D_2_ at a comparable or even greater rate than previously reported at 2 °C and pH 5.5. However, the loading of DHA was less efficient under the same parameters and significantly different depending on whether the component was free or bound to triglycerides. Additional trials are required to optimise the encapsulation of DHA. Furthermore, they demonstrated that the addition of whey proteins and applying heat (85 °C/2 min) resulted in a significant decreased vitamin D_2_ in the micellar phase compared to unheated samples. However, vitamin D_2_ concentrations in the micellar phase were steady throughout the four days storage period. Further studies are needed to evaluate DHA encapsulation and lipophilic substances’ location within casein micelles. Casein micelle has also been used to encapsulate sambiloto (an herbal anti-diabetic drug). In simulated gastric fluid, casein micelles degraded rapidly to release the active components [173].

Crosslinking of casein micelles [110], was used to create casein nanoparticles using transglutaminase by pushing out micellar calcium phosphate. Recently, Yang and co-workers [134], investigated the microencapsulation of emodin, having antibacterial and potent antioxidant properties, in micellar casein by applying heat (25 °C, 30 °C, 37 °C) and ultrasound (20 kHz). Fluorescence evaluation revealed that the hydrophobic forces are the primary interaction between micellar casein and emodin while heating independent of ultrasounds. However, ultrasound generated acoustic cavitation, resulted in three times greater binding constant (ka) of casein–emodin complexes than heat treatment alone. This ka value was close to the previously reported value of binding emodin and βLG or tyrosinase and β casein and epigallocatechin [174,175].

Microencapsulation of emodin (10 µmol/g casein) by spray-drying revealed that casein micelles structure remain unchanged by heating with/without the presence of ultrasound, but antioxidant properties enhanced due to change in interaction domains with the application of ultrasound. Moreover, ultrasound exhibited a slow release of emodin in gastric environments by weakening the lactose and casein interaction and enhancing emodin deep inside casein micelles [134]. However, further research is needed to evaluate emodin–casein monomer interactions at different sonication conditions and emodin concentrations.

Most recently, poorly soluble drug celecoxib (CLXB) has been loaded in different casein formulations (casein nanoparticles CNP, recombined casein micelles r-CM, nanocapsules prepared from soy lecithin CNC) to find an appropriate carrier to enhance the solubility of poorly soluble celecoxib. Higher encapsulation efficiency and drug entrapment efficiency (90%) and dissolution efficiency were seen in CNP verses r-CM and CNC. The research concludes that the CNP could act as a cryoprotectant and lyophilise itself, though it can retain its structural integrity upon resuspension [176].

Casein micelles can be used as target-oriented hydrophobic bioactive carrier systems to provide additional health benefits for foods. For their use as transportation, vehicles provide different functional properties for encapsulating molecules, such as hydrophobic chemical binding molecules, surface activity, aggregation, gelation and contact with other polymers. However, different processing parameters, pH and temperature might affect casein micelles structure and encapsulation efficiency. There are still unknown areas to be explored, like physicochemical and morphological characteristics of casein micelles at various temperature and pH conditions.

### 4.2. Caseins and Nano Emulsions

Caseins and casein micelles are the most prevalent amphiphilic proteins that are widely used to make stabilised emulsions. Caseins can adsorb at the oil–water interface, thus having a high surface activity during homogenisation, processing and storage by preventing coalescence in emulsions under different conditions, such as pH, temperature, structure elasticity and aggregation [34]. Because of these properties, casein is now used to deliver different hydrophobic bioactive in emulsion-based drug delivery systems [6]. Moreover, their highly stable nature makes them suitable for stabilizing the emulsion. Caseinates have demonstrated more significant surface activity than casein micelles, though calcium caseinate has a higher protein content on the emulsion surface than sodium caseinates [177]. Moreover, multilayered casein-based delivery systems are also readily available in the nutraceutical industry [133]. For example, oil droplets rich in ω-3 are coated by caseinates and caseinates plus lactoferrin, which have more stability to pH change and salt addition due to crosslinking (among caseinate and lactoferrin) as compared to caseinate alone.

Casein also suppresses lipid oxidation in the emulsion, thus affecting the oxidative stability of the emulsions. Researchers [178], also found that antioxidant properties of caseins are higher than that of whey protein in linoleic acid emulsions for broader droplet size. However, the antioxidant impact of protein in emulsions counteracts the emulsion droplet size and protein form at higher protein concentrations in a continuous process. Sodium caseinate (SC) is a food-grade dairy emulsifier of proteins commonly found in the food industry compared to other proteins. SC can form a thicker, spherically stabilising film on the emulsion droplet interface that shields newly formed droplets from impurities, flocculation and coalescence [179], determined the physical stability of β-carotene (β-CE) in oil-in-water sodium caseinate nanoemulsion at various pH (2, 4, 6, 8, 10), temperature (80 °C), ion strength (0–500 mM) and time of storage (25 days). It was assessed that β-CE nanoemulsion is susceptible to aggregation at *Ip* pH of the sodium caseinate (pH 4–5), showed physical stability at 80 °C for 90 min and at ion strength (100–500 mmol/L). Overall, this research was significant for the encapsulation of carotenoids and other bioactive lipophilic agents to regulate emulsions’ stability by using sodium caseinate.

Encapsulation of β-carotene was also done in multilayered emulsion prepared by sodium caseinate and κ-carrageenan at pH 7 and 3.5. The higher amount of κ-carrageenan (1.5%) leads to microbeads’ formation with greater size and more spherical shape at both pH values. Microbeads produced at pH 3.5 and pH 7 had similar aspect ratios, but at pH 3.5, the microbeads had greater particle diameters since protein and polysaccharide are negatively charged at pH 7.0 and protein is positively charged at pH 3.5, preferring electrostatic interactions with the polysaccharide. As a result, at pH 3.5, κ-carrageenan was electrostatically bound to sodium caseinate at the oil surface, resulted in lesser free polysaccharide in solution and potentially slower gelation relative to microbeads formed at neutral pH. This presumably contributed to the greater breakdown of particles before gelation. On the other hand, the pH value has no significant effect on the morphology, encapsulation efficiency and suspension rheology of microbeads. The selected microbeads showed a monomodal distribution of sizes with a median varying diameter ranging from 130 to 160 µm. However, further studies are required to investigate sodium caseinate and κ-carrageenan interfacial layer capability to protect β-carotene while storage [143].

Mora-Gutierrez hypothesised that resveratrol and polysorbate-20 could bind to bovine and caprine casein, which may preserve resveratrol’s biological properties. The resveratrol–casein complex is stabilised by hydrophobic and hydrogen bonding as resveratrol binds to tryptophan residues of casein. Moreover, RP-HPLC results showed that resveratrol’s sensitivity to oxidative and thermal stresses accelerated by a large surface area of oil droplet size that reduced size. Besides, emulsifier usage influenced resveratrol’s chemical stability by protecting these types of hydrophobic phytochemicals by hydrophobic milk protein domains along with surfactant. However, a caprine–surfactant combination has been found ideal over bovine–surfactant complex in stabilising nanoemulsion incorporated by resveratrol. Further studies to describe the chemical degradation of resveratrol in nanoemulsion distribution systems stabilised by emulsifiers (caprine casein, polysorbate-20) and hydrocolloids (polysaccharides) are highly necessary; they can alter the structure and composition of the adsorbed stabilising substrate, with significant consequences for texture and shelf life [180]. Casein complexes with polysaccharides is an emerging interest in developing novel emulsifiers and stabilisers as casein can adsorb on the surface of oil–water emulsion.

In contrast, polysaccharides behave as emulsion stabilisers by forming an extended network and changing the viscoelastic properties. Caseins lost their emulsifying properties at Ip (pH 4.6) and follucolates; however, casein with polysaccharides could enhance the emulsion stability [142,181]. For example, the casein–xanthan gum complexes stabilised emulsion over a broader pH range and exhibit better ionic strength resistance than casein or gum alone [128]. The emulsion stability could be enhanced by broadening the pH range (2.5–6.5) with chitosan casein complexes as an alternative to casein. Chitosan, a polysaccharide, exhibits pH-responsive behaviour, has an effective viscosity enhancer even at acidic pH [182].

Casein micelles can be adsorbed strongly at the oil–water interface, showing high surface activity in response to different processing conditions. Hence, casein and casein micelles being amphiphilic and having structural flexibility are used in emulsion-based drug delivery systems to deliver several hydrophobic bioactives.

### 4.3. Casein-Based Complexes as Delivery Systems

Casein, being biodegradable and non-toxic, has attained interest to be used as a natural polymer. Its interaction with other polymers is an innovative way to use it as a delivery vehicle for sensitive food bioactive components in functional foods and nutraceuticals. Possible copolymers are casein and pectin, casein and dextran, caseinate and glucose, hydrolysed sodium caseinate and maltodextrin. Previously, extraction of casein-based complexes from milk chocolate and their availability as natural phosphorylation polymer make casein an excellent candidate to be used in the chocolate industry. Caseins (α, β, κ) are isolated from chocolate, complexed with phenolic compounds like p-coumaric acid and protocatechuic acid, by incubating at 55 °C, which induces stabilisation in the dairy chocolate and imparts a creamy taste. The casein–phenolic complexes are formed by five types of potential interaction; hydrogen bonding, hydrophobic interaction, π-bonding, covalent and ionic interaction, thus adding more stabilization to the food product. Covalent attachment exists with βLg, while non-covalent associations exist between casein and cocoa polyphenols [183].

It has also been stated that casein interactions with polyphenols alter the conformation of caseins, resulting in a decrease in the number of α helices and β sheets [184], so in a casein–polyphenol mixture, the antioxidant activity decreased slightly, indicating a major influence of casein on polyphenol activity. This reduction was more evident in casein that had been incubated with catechin or epicatechin. However, MALDI-TOF mass spectra of incubated caseins did not reveal any stable adduct between the individual caseins, neither with catechin/epicatechin nor with cocoa polyphenols derived from cocoa [184].

Casein and phenolic acids (protocatechuic acid or p-Coumaric acid from chocolate) interaction have been studied by Zhou and his colleagues by native PAGE. They observed three main bands (A, B, C) for α, β, κ caseins on the SDS-PAGE electrophoretogram for casein and casein incubated with protocatechuic acid and p-Coumaric acid. The casein–protocatechuic acid complex exhibited a reduction in the intensities of the α, β, κ caseins bands as well as the presence of three slower migration bands which are casein aggregation bands. These casein aggregation bands were not significantly different between casein control and casein—p-Coumaric acid than in casein–protocatechuic acid, thus indicating that protocatechuic acid favoured casein subunit aggregation through protein and phenolic associations, while the bigger and more hydrophobic phenolic components have a better affinity for proteins [185]. Additionally, in future studies, sufficient spectrometric mass methods will uncover systemic knowledge enhancement of casein–phenolic relationships in problematic foods systems.

Researchers have studied the complex formation between sodium caseinate, a protein isolate from whey, and hydrophobic lutein (found in carotenoids and grouped as xanthophyll) [186]. However, lutein is highly unstable under light, heat and varying oxygen conditions. These properties limit the use of lutein in the nutraceutical and food industries. Lutein binds with sodium caseinate (SC) and whey proteins (WPI) by hydrophobic interactions; however, the binding constant between whey protein isolates and lutein and between caseinate and lutein, prepared in alcoholic phase, remained bigger than prepared in phosphate buffer (PB). This was ascribed to the fact that large lutein aggregates developed in phosphate buffer before protein binding. Furthermore, the binding constants of sodium caseinate and lutein complexes were larger than those of whey protein isolates and lutein complexes, indicating that sodium caseinate has a stronger affinity for hydrophobic bioactive compounds than WPI does. Moreover, lutein’s chemical stability was better in sodium caseinate lutein complexes than in whey protein lutein complexes. Additionally, no change in the secondary structure composition of both milk proteins using software analysis of far CD protein spectra has been observed, demonstrating that lutein did not affect the secondary structure of either milk protein [187]. Thus, the hypothesis that the sodium caseinate aids in the stabilisation of bioactive components in the production of low-fat functional foods is supported.

In developing casein-based complexes as delivery vehicles for polyphenols, another study was conducted using β caseins and micelles. β caseins are considered a natural vehicle for the transportation of polyphenols, fat-soluble vitamins and water-insoluble drugs due to their self-assembling nature and binding properties. Cheng and colleagues were first investigating the interaction between β casein and cis/trans-resveratrol to form protein–polyphenol complexes. Results suggested the simultaneous binding of both resveratrol isomers (cis, trans) by forming protein–ligand complexes. However, trans-resveratrol’s encapsulation efficiency (69%) was more than cis-resveratrol (57%) due to the lower affinity of cis-isomer with β casein. Moreover, resveratrol’s addition does not affect the size and ζ potential of β casein [138].

Xu and colleagues [142] produced ternary nanoparticles (by hydrophobic and electrostatic interactions) by the combinations of casein (CN), curcumin (CUR) and soy soluble polysaccharides (SSPS) utilisation of alkaline dissolution, by applying high-pressure homogenisation (800 bar/4 min) and by the acidification. While dropping pH 10–4, both CN and SSPS could form complexes and CUR could be encapsulated in self-assembled CN, being insoluble in acidic pH. Then again, CN loading efficiency decreased by increasing CUR concentration at acidic pH, which could be resolved by applying high-pressure homogenisation, which ultimately breaks the non-covalent bonds present in self-aggregates to promote complex ternary formation. The ternary complex (CUR/CS/SSPS) has increased the CUR bioavailability by 3.4 folds in mice compared to CUR/tween-20. Hence, complex ternary nanoparticles have an effective system for curcumin loading, protection and bioavailability that further gives insight into the delivery of food components insoluble at acidic pH [138].

All these findings offered help for future utilisation of casein micelles to make complexes with other polysaccharides/lutein/resveratrol to enhance their emulsifying and stabilising properties to acts as a carrier for polyphenols.

### 4.4. Casein Micelles as Hydrogels

Caseins micelles are also widely used as hydrogels for the delivery system in the food and drug industries. Hydrogels consist of three-dimensional structures that are water-soluble polymers and can trap and retain water molecules inside them. In the food industry, the porous structures of casein micelles make them choose substances as they can be synthesised using a wide range of food-grade polymers and effectively deliver other biomolecules within the cell system [188]. Additionally, they are readily available and have low preparation costs, hence making them feasible to use in drugs and other industries for water retention as compared to collagen, albumins from bovine serum [189], polypeptides or other materials [190] commonly used to prepare the hydrogels that are proteinaceous. Few studies have reported protein-based casein hydrogels’ preparation by crosslinking microbial transglutaminase [131], to bind to the drug molecules and targeted and controlled the release of drugs. For example, the researchers Li and colleagues studied the gelation kinetics and properties induced into composite hydrogels, particularly the involvement of aldehyde groups in producing oxidized hyaluronic acid. The researchers also pioneered in oxidising hyaluronic acid and crosslinking the casein proteins in water environments [181]. The purpose has been to use the polymer in the drug industry and determine the cytotoxic and drug retention and release behaviours in in vitro environments.

Profoundly stretchable and score harsh hydrogel has been created utilising casein micelles and polyacrylamide [148]. Acidification of the medium was essential to advance casein micelle proximation, which happened because of the neutral charge. This prompted the debilitating electrostatic repulsions between casein micelles. The formulated hydrogels were stretchable to more than multiple times their different length. Hydrogel score affectability was resolved for the hydrogels by setting indents of fluctuated sizes over each, at that point extending them to test their capacity to come back to their different sizes. The indents were watched coming back to the first sizes significantly after the hydrogel had been extended multiple times longer than its extraordinary length. The researchers likewise compressed the hydrogels, planned to utilise casein micelles crosslinked with polyacrylamide that returned to their unique shapes after pressure, while hydrogels defined utilising just casein micelles and just polyacrylamide were weaker and did not return to their unique shapes. Casein micelles friction and deformations were given as potential clarifications. The author additionally propose that the casein micelles may assimilate and spread the pressure applied to the polyacrylamide arrangement that keeps up the hydrogel structure [30].

Casein gels have been prepared by adjusting the pH from 9 to 1 to encapsulate caffeine by spray-drying in the absence of any crosslinking agents (genipin) because the gelled caseins have similar properties to crosslinked caseins without being toxic [136]. They evaluated that caffeine has been released in a regulated manner from casein gel tablets, with release times ranging from a few minutes to several days. The casein gel, which resembles shampoo or lotion, might be spray-dried and manufactured into elastic casein tablets. According to DSC and FTIR tests, the casein gel has not been denatured. The optimal intake temperature for spray-drying casein–coffee mixes is determined to be 150 °C. The pressure-controlled release was carried out at compression pressures of 160, 80 and 8 MPa (with excipient), resulting in caffeine concentrations greater than 80% within about 24, 12 and 3–6 h, respectively. By adjusting the coffee–casein ratio between 0.1 and 1, the release time for caffeine at an 80 per cent concentration may be adjusted between 12 and 0.5 h. The spray-dried casein–coffee particles had comparable morphologies, with mean diameters of around 10 µm and raisin-like forms. FTIR research revealed no significant interactions between the casein and coffee components [136].

Spray-dried and oven-dried casein gels have also been formulated to encapsulate caffeine, acetaminophen and vitamin C to investigate its controlled release under various fabricated methods. Afterwards, tablets were made by compression at 160 MPa of each spray-dried microencapsulated particles to investigate their release rate in SGF at 37 °C. The time necessary to release 80% of the contents from milk protein tablets is highest in casein–acetaminophen, decreasing gradually from casein–coffee to casein–ascorbic acid and lowest in the case of WPI–acetaminophen. Acetaminophen is more resistant to heat than coffee and ascorbic acid and has been used to investigate heat-enhanced controlled release. FTIR and DSC were used to investigate the denaturation (heat-gelation) of milk proteins. WPI is less heat stable than casein. After heating, the milk protein tablets containing acetaminophen release at a slower pace. The 12 h release of acetaminophen from casein and casein–WPI tablets is almost lowered from 80% to 70% (a 10% reduction) and from 90% to 80%, respectively. Only 80% of the acetaminophen in heated casein–acetaminophen tablets is released within 24 h, and the release duration is over two days. Using food materials, this study establishes an effective mechanism for the regulated release of food and medicinal substances [137].

**Figure 4:**
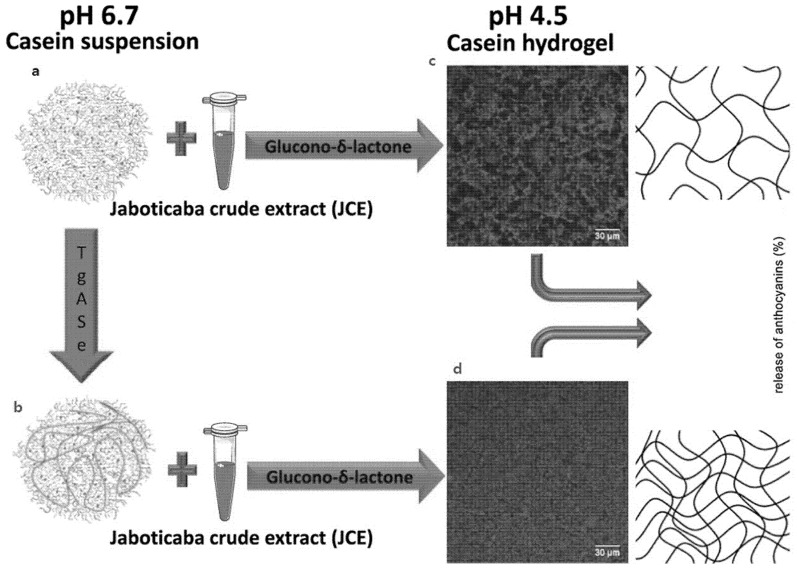
(**a**) Casein micelles; (**b**) Casein micelles plus added transglutaminase; (**c**) Casein hydrogel and added jaboticaba extract but no transglutaminase; (**d**) Casein hydrogel with added transglutaminase and jaboticaba extract. Reproduced with permission from [Nascimento, L.G.L.; Casanova, F.; Silva, N.F.N.; Teixeira, Á.V.N.d.C.; Júnior, P.P.d.S.P.; Vidigal, M.C.T.R.; Stringheta, P.C.; Carvalho, A.F.], [Use of a crosslinked casein micelle hydrogel as a carrier for jaboticaba (*Myrciaria cauliflora*) extract], [Food Hydrocoll.], (2020).

Another recent study [28] has indicated that crosslinked casein micelle could be utilised to transport and release jabuticaba extract (JE). The authors revealed earlier that hydrophobic interactions dominated at pH 2, but electrostatic interactions sensitive to ionic strength were the major binding forces at pH 7 [191]. The goal of the work was the production of a hydrogel by acid gelation from crosslinked casein micelles that was capable of storing and releasing Jabuticaba extract under regulated conditions (JE). Additionally, the JE release profiles at pH 2.0, 4.5 and 7.0 were studied. The CMs suspension pH was adjusted to 6.7 and Transglutaminase (Tgase) was added to CMs. Transglutaminase usage could balance the pace of liberation. No effect has been found on the structure of casein micelles in suspension, whereas Tgase promotes the creation of a denser and more homogenous network in casein hydrogel samples (Figure 4).

Because it catalyses the creation of covalent bonds within the protein matrix, the Tgase activity resulted in increased protein-protein interaction. The existence of the crosslinked frameworks indicated the slower release of jabuticaba extract at all pH levels, with a most exciting release at pH 7 and least at pH 2. At all pH levels studied, the Tgase-treated hydrogel samples released at a slower pace than the untreated hydrogel samples (Figure 4). Further investigations under simulated and in vivo digestion conditions must be done to decide casein micelle hydrogels’ capacity to shield JE and perhaps raise its bioavailability.

Finally, casein micelle has been considered ideal for producing hydrogels in functional foods and nutraceuticals due to their non-toxic, hydrophilic and biocompatible behaviour and reactive site availability for chemical modifications. However, research gaps exist in determining these products’ potential in terms of use at the industrial scale.

## 5. Conclusions and Future Challenges

In conclusion, this review provides a comprehensive overview of casein micelles regarding their composition, properties and usage as targeted delivery systems for a variety of bioactive substances to provide food with additional health benefits. Owing highly surface-active and emulsifying properties, self-assembling nature and distinctive binding abilities, casein micelles act as a promising candidate to deliver bioactives in functional foods and nutraceuticals. Complexes of casein micelles with polysaccharides/gums/pectin/polymers to encapsulate hydrophobic compounds has also made significant progress in the last few years. This may be accomplished by lowering the pH since acidic pH causes caseins to obtain a positive charge, which interacts with the negative charge on polysaccharides. Furthermore, hydrophobic regions of casein micelles interact with other hydrophobic substances to produce nanoencapsules. Casein micelles, being amphiphillic exhibits excellent surface active and emulsifying properties to stabilise emulsions and their highly flexible conformation made them ideal for emulsion based delivery systems.

Casein micelles are also a great choice for smart hydrogels due to its intrinsic features, that release biomolecules in a variety of settings. Additionally, the water absorption and swelling properties of casein-based hydrogels enable the entrapment of specific molecules and their controlled release. Although various novel advancements in employing casein micelles as an effective delivery mode of bioactives have been reported in recent years, more comprehensive studies are required to increase the understanding of how exactly casein micelles to be used as a particular delivery system to deliver bioactives with numerous physical, chemical and structural properties.

Much of the current literature has worked on bioavailability of these bioactive components using cell line models or simple animal models. Thus, it is essential to carry out human clinical trials to investigate the full benefits of using casein micelles as a delivery system for bioactives. In addition, the development of functional foods designed for personalised health is a hot topic nowadays due to various health conditions and allergy conditions that people possess with the consumption of dairy proteins. So, having a though investigation of the optimal benefits in terms of bioavailability and bio functionality of various bioactive ingredients encapsulated within casein micelle delivery systems under specific human health and allergy conditions will increase the usage to the wider community. A full consideration of the regulatory status of these functional systems is a must as this will be consumed by both adults and children.

Use of non-thermal technologies such as high-pressure processing, pulsed electric field, ultrasound and UV light has attained considerable attention within the food industry recently due to their non-destructive, cost effective and energy efficient nature. Many studies have found that the use of these non-thermal technologies on casein micelles changes the casein micelle structure based on the processing conditions. However, how these structural changes obtained through non-thermal technologies are being utilised towards the efficient delivery of various bioactives is under-explored. So, it is quite important to research on how to utilise that unique structural change towards the delivery of either hydrophobic or hydrophilic bioactives.

Another significant challenge of using casein micelles as a delivery system is the production capabilities. Most of the studies up to date have focused mainly in lab scale. So, the question is how these results can get replicated when manufactured under pilot scale or large-scale operations. The controlling of conditions is difficult in some instance under large scale operational conditions. For instance, a lab scale experiment may have used a water bath for heating purposes, but large-scale operations may have to use a heat exchanger. Thus, how, under these conditions, does the casein micelle structure behave, can they be replicated, and is the bioactive delivery the same, are some questions that need to be addressed in depth.

## Figures and Tables

**Table 1:** Comparison of distinct properties of α_S1_, α_S2_, β and κ Caseins.

Characteristics	Caseins Types			
	α_S1_	α_S2_	β	κ
Natural conformation	Unfolded structure	Unfolded structure	Unfolded structure	Unfolded structure
Percentage in milk	1.2–1.5	0.3–0.4	0.9–1.1	0.3–0.4
Amino acid residues = Hydrophilic peptides + Hydrophobic regions	199 = 63 + 136	207 = 118 + 89	209 = 42 + 167	169 = 64 + 105
Molecular weight (Da)	23,000	25,000	24,000	19,000
Setting in milk at room temperature	Inside Micelle	Inside Micelle	Inside Micelle	Micelle surface
Number of proline resides/number of cysteine residues	17/0	10/2	34–35/0	20/2
No. of Phosphate groups	8	10–13	5	1
No. of S-S groups/No of S-H groups	0/0	1/0	0/0	1/0
Analyzed charge at pH 6.6 (mV)	(−21)–(−23.5)	(−12.2)–(−17.1)	(−11.8)–(−13.8)	(−2.0)–(−3.0)
primary structure IP versus IP after phosphorylation	4.91/4.42	8.34/4.95	5.13/4.65	5.93/5.6

Reproduced with permission from [Rehan, F.; Ahemad, N.; Gupta, M.], [Casein nanomicelle as an emerging biomaterial], (2019); [Głąb, T.K.; Boratyński, J.], [Potential of Casein as a Carrier for Biologically Active Agents], [Top. Curr. Chem.], (2017); [Ranadheera, C.; Liyanaarachchi, W.; Chandrapala, J.; Dissanayake, M.; Vasiljevic, T.], [Utilizing unique properties of caseins and the casein micelle for delivery of sensitive food ingredients and bioactives], [Trends Food Sci. Technol.], (2016).

**Table 2:** Intrinsic and Extrinsic Factors to Modify Casein Micelles Structure and Functionalities.

Physical Methods	Biochemical Effect	Charge on Casein	Chemical Methods	Biochemical Effect	Charge on Casein	Enzymatic Methods	Biochemical Effect	Charge on Casein
Temperature (High) [42,84,86] Temperature (Low) [87,88]	Blockage of lysyl residues by lactose β-lactoglobulin covalent association Calcium phosphate precipitation and solubilisation Β casein solubilisation	Reduced negative charge Not determined	Reaction with sugar Glycation [90] Lactosylation [91]	Blockage of lysyl residues Blockage of lysyl residues	More negative More negative	Dephosphorylation [92,93]	organic phosphate removal from phosphoseryl residues	Reduced negativity
pH (Acid) [94,95,96,97] pH (alkaline) [98,99]	Protonation of casein Decrease of cations casein interactions Increase of the casein ionisation Insolubilisation of calcium phosphate	Reduced negativity More negative	Chemical Reticulation [100,101]	Blockage of lysyl residues	More negative	Deamidation [102,103]	- Release of ammonia from glutamine transformed into glutamic residues	More negative
Pressure [104,105,106,107]	Casein micelles distruptions	Not determined	Phosphorylation [108]			Reticulation [109,110]	Lysyl and glutamine crosslinking	Enhanced negativity
Ultrasound [20,111,112,113]	Casein micelles disruptions	Not determined	Glycosylation			Deglycosylation [114,115]	- Release of NANA	No effect
Addition of cations (di & trivalent) [116,117]	Direct association of added cation to casein Association of cation-inorganic phosphate to casein micelles Increase in ionic strength	Less negative	Succinylation [103] Acetylation [118]	Lysyl residues inhibition	More negative More negative	Proteolysis [119,120]	- Release of caseino macropeptide negatively charged between 106 to 169 peptides	Reduced negativity between 1–105 peptides
Adding salt [121,122]	Micellar calcium solubilisation Ionic strength enhancement	No change						
Removal of diffusible ions	Diffusible ions removal	More negative ions						
Calcium chelatants addition [123,124]	Casein and calcium association reductions Micellar calcium phosphate solubilisation	More negative ions						
External ligands addition [125]	Hydrophobic and hydrogen interactions to caseins	ND						

**Table 3:** Casein Micelles-Based Capsules and Hydrogels in Delivering Food Bioactives.

Casein Type	The Technique Used for Preparing Loaded Reassembled Casein Micelles	Bioactive	Encapsulation Mechanism	References
Micellar casein	• Casein–emodin complex formation by vortex • Heat and Ultrasound treatments • Spray-drying microencapsulation • In Vitro digestion evaluation	Emodin	Microencapsulation	[134]
β casein micelle	• Drug loaded β caseins dispersion • Freeze drying • Making and description of gastro-resistant Nanoparticle in Microparticle Delivery Systems • pH 2 and 6.5 • In Vitro drug release	Antiretroviral (ARV) combinations of Darunavir, efavirenz and ritonavir encapsulation in β caseins and further within Eudragit L100	Co-encapsulation, Nanoparticle-in-microparticle delivery system (NiMDS)	[135]
Casein gels	• Casein gel production at pH 1 and 9 • Spray-dried gel and tablet • Oven-dried gel and tablets • Controlled release under various compression methods	Caffeine	Gels	[136,137]
β casein micelle Sodium Caseinate	• β casein preparation in 7.4 phosphate buffer • Blending of protein and resveratrol • Production of polysaccharide conjugates by Millard reaction Resveratrol loading at pH 7.5	Resveratrol	Encapsulation Emulsions	[138,139,140]
β casein depleted Casein micelles	• Centrifugation • Lyophilisation • Mixing by shaker • Ultracentrifugation • Enzymatic crosslinking	Linoleic acid	Nanoencapsulation	[141]
Caseins	• Acidification • Homogenisation at high pressure • Curcumin/casein/soy polysaccharide complex at pH 10.0 • In Vitro digestion evaluation • CUR pharmacokinetics of CUR/CN/SSPS in mice	Curcumin	Nanoencapsulation	[142]
Casein Micelle	• Chemical acidification • Crosslinking by transglutaminase	Jaboticaba extract	Hydrogels	[28]
Sodium casienate/Carrageenan	• Primary and multilayered emulsion preparations • Microbeads preparation by gelation in an atomiser	β carotene	Emulsions/Gels	[143]
Casein micelles	• Mineral arrangement restoration and spray-drying • Homogenisation at high pressure • pH and temperature-induced opening	β carotene	Nanoencapsulation	[25,144,145,146]
Re-assembled casein micelles (r-CM) Sodium caseinate (CNP)	• Binding at pH 7.4 and temperature 74 °C • Centrifugation • EGGC binding r-CM and CNP • Encapsulation efficiency determination	Epigallocatechin gallate (EGGC), folic acid	Nanoencapsulation	[147]
Casein micelles	• Preparation of casein-PAAm hydrogels by free radical polymerisation	Polyacrylamide	Hydrogels	[148]
Casein micelles	• Spray-drying pH-shifting • High-pressure treatment	curcumin	Nanoencapsulation	[149,150,151,152,153,154]
Reassembled Casein micelles	• Restoration of mineral composition and ultrahigh-pressure homogenisation	Vitamin D_3_	Nanoencapsulation	[145,146,155,156,157]
Micellar Casein	• A shift in pH and ultrasonication	Fish oil	Emulsions	[158]
Micellar casei Re-assembled casein micelle from micellar casein	• A shift in pH and ultrasonication	Vegetable oil (Lactobacillus and Bifidobacteria	Nanoencapsulation Microencapsulation	[158,159]
Casein micelles	• Mineral composition restoration • Homogenisation with high pressure	Omega-3	Nanoencapsulation	[158]
β Casein micelles	• Lyophilization	Celecoxib	Nanoencapsulation	[160]
Casein micelles + konjac glucomannan (KGM)	• Enzyme-induced casein KGM hydrogels preparation • Ageing in refrigeration	Docetaxel	Gel	[161]
Casein micelles	• Skim milk natural conditions • Thermally treated commercial skim milk	Vitamin A	Nanoencapsulation	[156,162]
Casein micelles	• Mineral composition restoration and homogenisation at high pressure • Re-assembly of casein micelles	Vitamin D_2_	Nanoencapsulation	[155]
Casein micelles		Rosemary Extract	Nanoencapsulation	[163]
Casein micelle		Lactoferrin	Nanoencapsulation	[164]
Casein micelle	• Spray-drying crosslinked with genipin	Alfuzosin	suspension	[165]
Casein micelle	• Spray-drying crosslinked with genipin	Flutamide	Microencapsulation	[165]

## Data Availability

Data availability statement has not been included in this review because it is based on previously published data.
